# Un cas d'endocardite brucellienne en Tunisie

**DOI:** 10.48327/mtsi.v5i2.2025.668

**Published:** 2025-04-03

**Authors:** Oussema HADDAR, Rania AMMAR, Mabrouk BAHLOUL, Chokri BEN HAMIDA

**Affiliations:** 1 Service de cardiologie, Centre hospitalier universitaire Hédi Chaker de Sfax, Tunisie; 2 Service de réanimation médicale du Centre hospitalier universitaire Habib Bourguiba de Sfax, Tunisie; 3 Université de Sfax, Tunisie

**Keywords:** Endocardite à *Brucella,*, Endocardite infectieuse, Endocardite bactérienne, Brucellose, Tunisie, Afrique du Nord, *Brucella* endocarditis, Infective endocarditis, Bacterial endocarditis, Brucellosis, Tunisia, North Africa

## Abstract

**Introduction:**

L'endocardite à *Brucella* (EB) est une complication rare mais grave de la brucellose, constituant la principale cause de mortalité liée à cette infection. Elle est souvent difficile à diagnostiquer en raison de ses symptômes non spécifiques et de sa faible prévalence.

**Observation:**

Ce cas clinique concerne un homme de 59 ans, porteur d'une prothèse aortique mécanique, présentant une insuffisance cardiaque avec désinsertion prothétique, abcès de l'anneau aortique et hémocultures négatives. Le diagnostic a été confirmé par des tests sérologiques et la culture de la pièce opératoire, révélant une infection à *B. melitensis* liée à la consommation de lait cru non pasteurisé. Le traitement a nécessité une intervention chirurgicale pour remplacer la prothèse et drainer l'abcès, ainsi qu'une antibiothérapie prolongée combinant doxycycline, cotrimoxazole et rifampicine. Malgré les complications postopératoires, l’évolution hémodynamique et infectieuse a été favorable.

**Conclusion:**

Ce cas met en évidence l'importance d'une approche multidisciplinaire, incluant des tests sérologiques ciblés et l'imagerie cardiaque, pour un diagnostic précoce. Il souligne également la nécessité d'une prise en charge chirurgicale associée à une antibiothérapie adaptée pour améliorer le pronostic des patients atteints d'EB.

## Abstract (en)

**Introduction:**

*Brucella* endocarditis (BE) is a rare but serious complication of brucellosis and is the leading cause of death from this infection. It is often difficult to diagnose due to its non-specific symptoms and low prevalence.

**Observation:**

This clinical case concerns a 59-year-old man with a mechanical aortic prosthesis who presented with heart failure with prosthetic disconnection, aortic annulus abscess, and negative blood cultures. The diagnosis was confirmed by serologic testing and culture of the surgical specimen, which revealed infection with *B. melitensis* associated with consumption of unpasteurized raw milk. Treatment required surgery to replace the prosthesis and drain the abscess, and prolonged antibiotic therapy with doxycycline, cotrimoxazole, and rifampicin. Despite postoperative complications, hemodynamic and infectious outcomes were favorable.

**Conclusion:**

This case highlights the importance of a multidisciplinary approach, including targeted serologic testing and cardiac imaging, for early diagnosis. It also emphasizes the need for surgical management combined with appropriate antibiotic therapy to improve the prognosis of patients with BE.

## Introduction

En Tunisie, la brucellose sévit toujours à l’état endémique et pose un problème de santé publique en recrudescence ces dernières années [5]. La complication la plus redoutable est l'endocardite à *Brucella* (EB) [1]. Elle est la cause la plus fréquente de décès [6, 9]. Le diagnostic de l'EB est souvent difficile en raison de sa présentation clinique non spécifique et du faible taux de prévalence (1,3 %-1,8 %) limitée à quelques cas cliniques [9, 11], ce qui retarde parfois la prise en charge thérapeutique appropriée [4]. La littérature ne rapporte que quelques cas d'EB sur valve mécanique [13]. Le traitement comporte une combinaison d'antibiothérapie avec un traitement chirurgical [12].

## Observation

**Figure 1:**
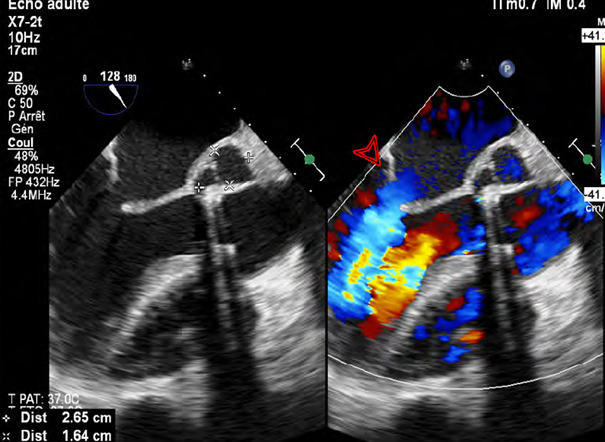
Échographie trans-œsophagienne post opératoire (coupe mi-œsophagienne 3 cavités 120 degrés) montrant une collection anéchogène post détersion chirurgicale de l'abcès de l'anneau

Mr A.M. est âgé de 59 ans, originaire de Kebelli, fermier, aux antécédents d'hypertension artérielle, et atteint de dyslipidémie. Il a été opéré pour remplacement de la valvule aortique par prothèse mécanique 20 ans auparavant. Il a été hospitalisé au service de cardiologie pour tableau d'insuffisance ventriculaire gauche avec à l'examen un souffle diastolique au foyer aortique. Une échographie cardiaque transthoracique a montré un ventricule gauche non dilaté hypertrophié avec une fonction systolique conservée à 60 %. La prothèse aortique n’était pas sténosante mais présentait une fuite importante du côté mitral sans image de végétation. Pour mieux qualifier cette fuite prothétique, une échographie transœsophagienne était faite montrant une désinsertion de prothèse en regard du trigone mitro-aortique avec abcédation et fuite importante. Le diagnostic d'endocardite infectieuse sur prothèse aortique compliquée de désinsertion de la prothèse était évoqué. Des séries d'hémocultures étaient réalisées, revenant négatives, et une série souillée par un *Staphylococcus* à coagulase négative. Une antibiothérapie probabiliste était instaurée à base de vancomycine 30 mg/kg et de ceftriaxone 2 g, 2 fois par jour. Le patient bénéficiait d'une chirurgie itérative « redux » avec remplacement de la prothèse aortique et mise à plat de l'abcès de l'anneau aortique (Fig. 1). Les suites opératoires immédiates étaient marquées par la survenue d'un bloc auriculoventriculaire complet à la sortie de la circulation extracorporelle, spontanément résolutif, suivi de passage en flutter à conduction variable rapide à 200/min, mal toléré, réduit par un choc électrique externe. L’évolution était marquée par un état de choc cardiogénique avec dysfonction sévère du ventricule gauche à 15 % et une hypokinésie globale. Une cure de lévosimendan était administrée avec une bonne évolution sur le plan hémodynamique et échographique (amélioration de la fonction d’éjection ventriculaire à 30, puis 40 %). Sur le plan infectieux, le patient restait fébrile avec installation d'un état de choc septique et aggravation du syndrome inflammatoire biologique. La culture de la pièce opératoire, ainsi que la sérologie de Wright (test de rose Bengale) étaient fortement positives à *B. melitensis* (1/160). L'antibiothérapie initiale était changée par l'association doxycycline, cotrimoxazole et rifampicine.

## Discussion

En Tunisie, la prévalence de l'EB a varié de 1,28 en 2003 à 8,94 pour 100 000 habitants en 2017 [5]. Dans la région du sud-ouest, comme pour le cas de notre patient originaire de Kebelli, le niveau d'endémicité le plus élevé dans le pays a été noté depuis 1995 avec des taux d'incidence toujours supérieurs à 20 cas pour 100 000 habitants par an [5]. Le diagnostic d'EB a été fait par l'interrogatoire, notamment la prise de lait cru non pasteurisé, l’échographie cardiaque ainsi que la culture et la sérologie spécifique de la brucellose. L'EB fait partie de l'endocardite infectieuse (EI) à hémoculture négative dans laquelle aucun microorganisme causal n'est isolé à l'aide des méthodes habituelles d'hémoculture [3, 7]. L'EI causée par des bacilles à Gram négatif non HACEK (espèces autres que *Haemophilus, Aggregatibacter, Cardiobacterium, Eikenella,* and *Kingella)* est rare [10]. Les humains se contaminent principalement par voie digestive ou cutanéomuqueuse. La contamination digestive par ingestion de lait cru ou de ses dérivés frais (fromage, lait caillé) provenant d'animaux infectés, de plus en plus fréquente, est devenue la principale voie de contamination [2], comme pour notre patient. Les EB surviennent habituellement sur une valvulopathie préalable, et intéressent la valve aortique dans 75 % des cas [8, 12]. L'EB peut se compliquer de façon fréquente d'un d'abcès myocardique jusqu’à 43 % des cas [6]. Le diagnostic de la brucellose est difficile. Il nécessite l'isolement de la bactérie à partir d’échantillons de sang ou de tissus [4]. La sérologie de Wright est un test de première ligne confirmant le diagnostic. Cependant, il peut être faussement positif ou négatif. Le titre d'agglutination de Wright varie entre 1/20 et 1 /160. Le seuil 1/80 est spécifique et sensible [3]. Le traitement recommandé est une chirurgie précoce avec une antibiothérapie prolongée entre 3 et 6 mois basée sur une combinaison bactéricides de doxycycline (200 mg/24 h) plus cotrimoxazole (960 mg/ 12 h) et rifampicine (300–600 mg/24 h) [1, 7, 11].

## Conclusion

Ce cas clinique illustre les défis diagnostiques posés pour le diagnostic de l'EB, en raison de la présentation souvent peu spécifique. Il met en lumière l'importance des tests sérologiques ciblés et de l'imagerie cardiaque pour détecter précocement les atteintes valvulaires. Ce diagnostic doit être évoqué en Tunisie en raison de l'endémicité de l'EB.

## Consentement éclairé

Nous avons obtenu le consentement oral du patient.

## Financement

Ce travail n'a bénéficié d'aucun financement.

## Remerciement

Dr. Olfa Gargouri, Dr. Imed Frikha

## Contributions des auteurs et de l'autrice

Oussema Haddar : conception du rapport de cas, prise en charge diagnostique et thérapeutique du patient, rédaction, révision et validation du tapuscrit.

Rania Ammar : prise en charge diagnostique et thérapeutique du patient, rédaction, révision et validation du tapuscrit.

Mabrouk Bahloul : rédaction, révision et validation du tapuscrit.

Chokri ben Hamida : rédaction, révision et validation du tapuscrit

## Conflits d'intérêts

Les auteurs ne déclarent aucun lien d'intérêts lié à ce travail.
