# Supplementary material for: A case of brucella endocarditis in Tunisia
Source: Med Trop Sante Int. 2025 Apr 3;5(2):mtsi.v5i2.2025.668. [Article in French] doi: 10.48327/mtsi.v5i2.2025.668 (PMC12308575; doi:10.48327/mtsi.v5i2.2025.668)
Supplement: Related texts

# Research article: A case of brucella endocarditis in Tunisia

## Introduction

In Tunisia, brucellosis is still endemic and has become a public health problem with an increase in recent years [5]. The most serious complication is *Brucella* endocarditis (BE) [1]. It is the leading cause of death [6, 9]. The diagnosis of BE is often difficult due to its non-specific clinical presentation and low prevalence rate (1.3%-1.8%) limited to a few clinical cases [9, 11], which sometimes delays appropriate therapeutic management [4]. Only a few cases of BE on a mechanical valve have been reported in the literature [13]. Treatment consists of a combination of antibiotic therapy and surgical treatment [12].

## Observations

Mr. A.M. is a 59-year-old farmer from Kebelli with a history of hypertension and dyslipidemia. He underwent aortic valve replacement surgery with a mechanical prosthesis 20 years ago. He was admitted to the cardiology department for left ventricular failure with a diastolic murmur at the aortic root. A transthoracic echocardiogram showed a non-dilated, hypertrophied left ventricle with preserved systolic function at 60%. The aortic prosthesis was not stenotic but showed significant leakage on the mitral side without vegetation. To better characterize this prosthetic leak, transesophageal echocardiography was performed, which showed disinsertion of the prosthesis opposite the mitral-aortic trigone with abscess formation and significant leak. A diagnosis of infective endocarditis of the aortic prosthesis complicated by prosthesis dislodgement was made. Blood cultures were obtained and were negative, and one culture was contaminated with a coagulase-negative *Staphylococcus.* Probabilistic antibiotic therapy was initiated with vancomycin 30 mg/kg and ceftriaxone 2 g twice daily. The patient underwent iterative surgery “redux” with replacement of the aortic prosthesis and drainage of the aortic annulus abscess (Fig. 1). The immediate postoperative period was characterized by the onset of complete atrioventricular block on withdrawal from the extracorporeal circulation, which resolved spontaneously, followed by poorly tolerated rapid variable conduction flutter at 200/min reduced by an external electric shock. The evolution was characterized by a state of cardiogenic shock with severe left ventricular dysfunction at 15% and global hypokinesia.

**Figure 1:** Post-operative trans-esophageal ultrasound (mid-esophageal section 3 cavities 120 degrees) showing an anechogenous collection after surgical debridement of the annulus abscess

A treatment with levosimendan was administered with a good evolution in terms of hemodynamics and echography (improvement of ventricular ejection function to 30% then 40%). The patient remained febrile with the onset of septic shock and worsening of the biologic inflammatory syndrome. The culture of the surgical specimen as well as Wright's serology (rose Bengal test) were strongly positive for *B. melitensis* (1/160). Initial antibiotic therapy was changed to doxycycline, cotrimoxazole, and rifampicin.

## Discussion

In Tunisia, the prevalence of BE has varied from 1.28 in 2003 to 8.94 per 100,000 inhabitants in 2017 [5]. In the southwestern region, as in the case of our patient from Kebelli, the highest endemicity level in the country has been observed since 1995, with incidence rates still above 20 cases per 100,000 inhabitants per year [5]. The diagnosis of brucellosis was made by interview, especially the consumption of unpasteurized raw milk, cardiac ultrasound, as well as culture and specific brucellosis serology. EB is a subset of blood culture-negative infective endocarditis (IE) in which the causative microorganism cannot be isolated by standard blood culture methods [3, 7]. IE caused by non-HACEK gram-negative bacilli (species other than *Haemophilus, Aggregatibacter, Cardiobacterium, Eikenella,* and *Kingella)* is rare [10]. Humans are primarily contaminated by the gastrointestinal or cutaneous-mucosal route. Gastrointestinal contamination by ingestion of raw milk or its fresh derivatives (cheese, curdled milk) from infected animals, which is increasingly common, has become the main route of contamination [2], as in the case of our patient. BEs usually occur in the setting of pre-existing valvulopathy and involve the aortic valve in 75% of cases [8, 12]. BE can often be complicated by a myocardial abscess in up to 43% of cases [6]. Brucellosis is difficult to diagnose. It requires isolation of the bacteria from blood or tissue samples [4]. Wright's agglutination test is a first-line test to confirm the diagnosis. However, it can be falsely positive or negative. Wright's agglutination titer varies between 1/20 and 1/160. The 1/80 threshold is specific and sensitive [3]. The recommended treatment is early surgery with prolonged antibiotic therapy between 3 and 6 months based on a bactericidal combination of doxycycline (200 mg/24 h) plus cotrimoxazole (960 mg/12 h) and rifampicin (300-600 mg/24 h) [1, 7, 11].

## Conclusion

This clinical case illustrates the diagnostic challenges posed by the often nonspecific presentation of BE. It highlights the importance of targeted serologic testing and cardiac imaging for early detection of valvular damage. This diagnosis should be considered in Tunisia due to the endemicity of BE.

## Informed consent

Verbal consent was obtained from the patient.

## Funding

This work did not receive any funding.

## Acknowledgements

Dr. Olfa Gargouri, Dr. Imed Frikha

## Authors’ contributions

Oussema Haddar: design of the case report, diagnostic and therapeutic management of the patient, drafting, revision and validation of the typescript.

Rania Ammar: diagnostic and therapeutic management of the patient, writing, revision and validation of the typescript.

Mabrouk Bahloul: drafting, revision and validation of the typescript.

Chokri ben Hamida: drafting, revision and validation of the typescript.

## Conflicts of interest

The authors declare that they have no competing interests.
